# 2778. Retrospective evaluation of effectiveness of Flomoxef for infections due to extended-spectrum β-lactamase-producing Enterobacterales

**DOI:** 10.1093/ofid/ofad500.2389

**Published:** 2023-11-27

**Authors:** Ryuji Koizumi, Kayoko Hayakawa, Yukihiro Hamada, Kazuhisa Mezaki, Norio Ohmagari

**Affiliations:** National Center for Global health and Medicine, Shinjuku-ku, Tokyo, Japan; National Center for Global Health and Medicine, Shinjuku-ku, Tokyo, Japan; Tokyo Women's Medical University, Tokyo, Tokyo, Japan; National Center for Global Health and Medicine, Shinjuku-ku, Tokyo, Japan; National Centre for Global Health and Medicine, Shinjuku, Tokyo, Japan

## Abstract

**Background:**

Flomoxef (FMOX), an oxacephem, is resistant to hydrolysis by extended-spectrum β-lactamase (ESBL), and is expected to serve as a carbapenem-sparing therapy for infections caused by ESBL-producing Enterobacterales (ESBLPE). FMOX has been approved and used in Japan for the treatment of ESBLPE infections; however, data on its effectiveness and optimal dosing are limited.

**Methods:**

This retrospective observational study was conducted at the National Center for Global Health and Medicine. We reviewed the medical records of patients with ESBLPE infections treated with FMOX for > 48 h, from 2019 to 2021. The time above the minimum inhibitory concentration (MIC) (TAM) of ESBLPE was calculated using the MIC value of FMOX for ESBLPE and the simulated FMOX concentration. Creatinine Clearance (CrCl) was calculated using the Cockcroft-Gault equation. Clinical effectiveness was defined as improvement in symptoms compared with baseline after FMOX treatment, as judged by infectious disease specialists. Microbiological effectiveness was defined as reduction of urine ESBLPE bacterial load to≤10^3 CFU/ml for urinary tract infections (UTI), or disappearance of ESBLPE for non-UTI.

**Results:**

Fourteen patients were included in the study: 11 patients had ESBL-producing *E. coli* as the causative organism and nine patients had UTI. Bacteremia accounted for 35.7% of all patients. The median age of the study cohort was 81 years (interquartile range [IQR], 67.75 – 91). Nine of the patients were female.

FMOX doses varied according to the CrCl category (Table). TAM was 100% in all but one case (CrCl 48.9 mL/min, MIC of FMOX=0.5 mg/L, FMOX 1g q 12h, TAM=88.9%). Of the 13 cases for which clinical effectiveness could be assessed, FMOX was effective in nine of ten (90%) UTIs and one of three (33.3%) non-UTIs.

Microbiological effectiveness was evaluated in only two cases (UTI and pneumonia, respectively), and FMOX was microbiologically effective in both.

Characteristics of patients who received FMOX against infection caused by ESBL-producing Enterobacterales (n = 14)

**Figure:**
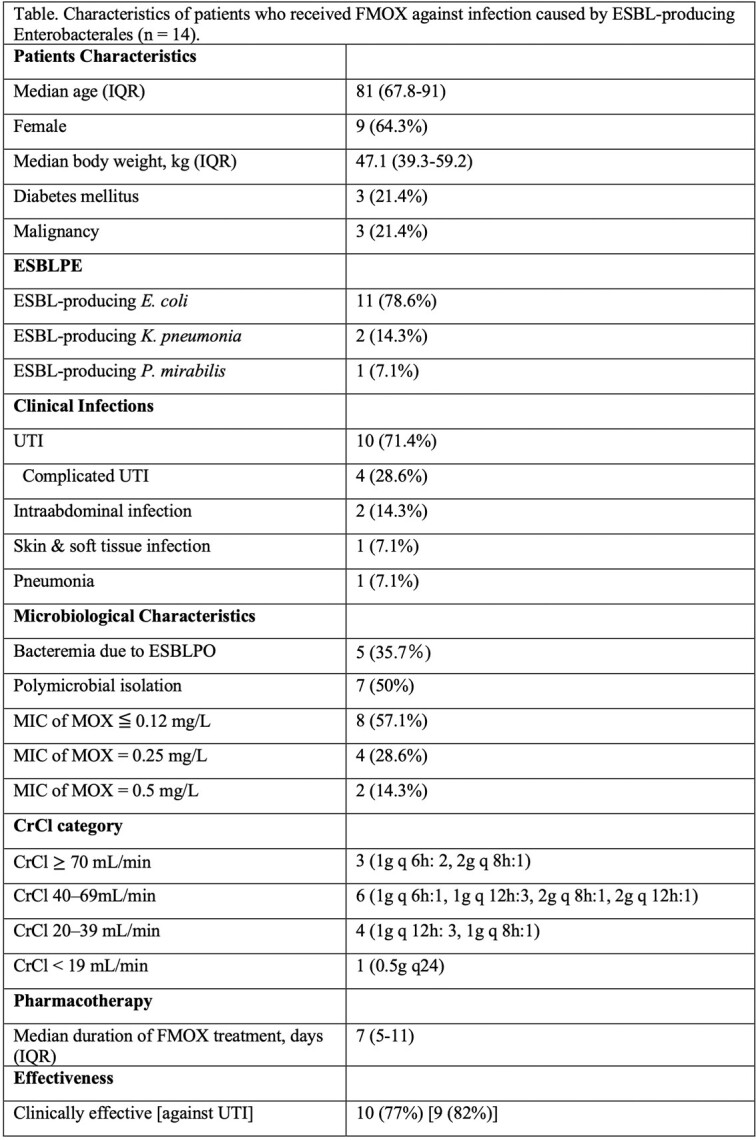

**Conclusion:**

FMOX was a clinically effective treatment for the majority of UTIs caused by ESBLPE.

For non-UTI patients, the rate of clinical effectiveness was low despite the high TAM.

FMOX is a potential carbapenem-sparing therapy for UTIs caused by ESBLPE.

**Disclosures:**

**All Authors**: No reported disclosures

